# Supplementary material for: Ras/MAPK Modifier Loci Revealed by eQTL in Caenorhabditis elegans
Source: G3 (Bethesda). 2017 Jul 27;7(9):3185–93. doi: 10.1534/g3.117.1120 (PMC5592943; doi:10.1534/g3.117.1120)
Supplement: Supplementary file 1 [file 3185FileS1.docx]

**Supplementary text:**

**Expression-QTL mapping using the FLP markers**

Using only the original FLP markers, only 60 out of 73 FLP markers met the two criteria explained in the materials and methods: (i) at least 15% representing the marker with one of the two genotypes and (ii) no strong linkage between chromosomes. Analysis of the statistical power of this reduced marker set showed that we can detect 79% of the eQTL explaining 40% of the variation of the QTL that segregate according to our markers. However, the power to detect QTL between the markers and the accuracy is reduced. Still, we detected 2292 genes (represented by 3181 array spots) with at least one eQTL (FDR=0.1, -log10(p)>3.1; **Table S1**). Of these genes, 1406 had a *cis-*eQTL and 886 had a *trans*-eQTL.

| **Table S1: eQTL mapped using the FLP genetic map** | | |
| --- | --- | --- |
|  | *cis*-eQTL | *trans*-eQTL |
| N2 higher | 987 (1454) | 480 (593) |
| CB4856 higher | 431 (596) | 421 (545) |
| Total^1^ | 1406 (2050) | 886 (1131) |
| ^1^: The discrepancy between the sum of N2 higher and CB4856 higher and the total is due to genes being represented by multiple spots, which often represent different splice variants. | | |

**FLP-based expression QTL across eQTL studies.**

The eQTL mapped based on the FLP markers were compared to the eQTL in other studies in non-sensitized RILs ([20](#_ENREF_20),[26-28](#_ENREF_26)). These datasets contain eQTL mapped over 9 different conditions (see Methods), which were compared at an FDR = 0.05 with the eQTL mapped in the sensitized miRILs (**Table S2**). Here we found that on average 73% of the genes with a *cis*-eQTL in the miRIL population also had a *cis*-eQTL in at least one other study. For the overlap in relation to effect direction (higher if the eQTL locus is N2 or CB4856), the same as for the full genetic map was found; the N2-higher eQTL showed a higher overlap (76%) compared to the CB4856-higher eQTL (65%).

The overlap in *trans*-eQTL – not taking location into account – showed the same pattern as in the full genetic map, on average 27% of the *trans*-eQTL were detected before, 2.7 times less than the overlap in *cis*-eQTL. Also here an effect of genotype was observed: 35% of the N2-higher eQTL were detected in previous studies, whereas only 17% of the CB4856-higher eQTL were detected before. Interestingly, this effect was less pronounced in the full genetic map, where we detected 94/448 (21%) of N2-higher and 134/464 (29%) of CB4856-higher genes with a *trans*-eQTL. It is likely that in the FLP map some *cis*-eQTL are mistaken for *trans*-eQTL and thereby result in the genotypic effect.

| **Table S2: overlap in eQTL mapped using the FLP map with other studies, overlap with at least one study is shown.** | | |
| --- | --- | --- |
|  | *cis*-eQTL (overlap/total, percentage) | *trans*-eQTL (overlap/total, percentage) |
| N2 higher | 746/987 (76%) | 168/480 (35%) |
| CB4856 higher | 280/431 (65%) | 72/421 (17%) |
| Total | 1026/1406 (73%) | 240/886 (27%) |
