# Supplementary figures and images for: Ras/MAPK Modifier Loci Revealed by eQTL in Caenorhabditis elegans
Source: G3 (Bethesda). 2017 Jul 27;7(9):3185–93. doi: 10.1534/g3.117.1120 (PMC5592943; doi:10.1534/g3.117.1120)

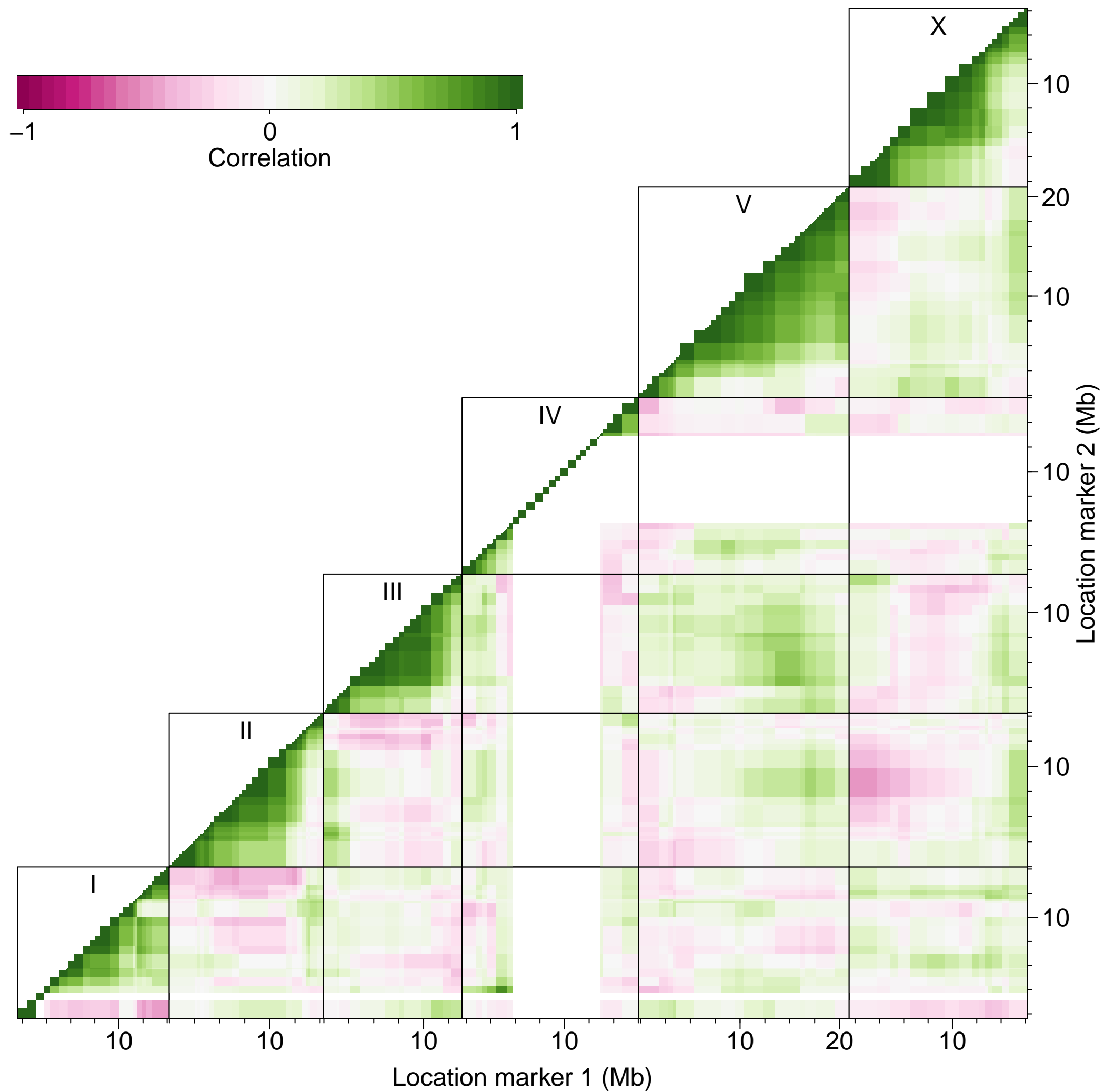

Supplement: Supplementary file 2 [file 3185FigureS1.pdf]

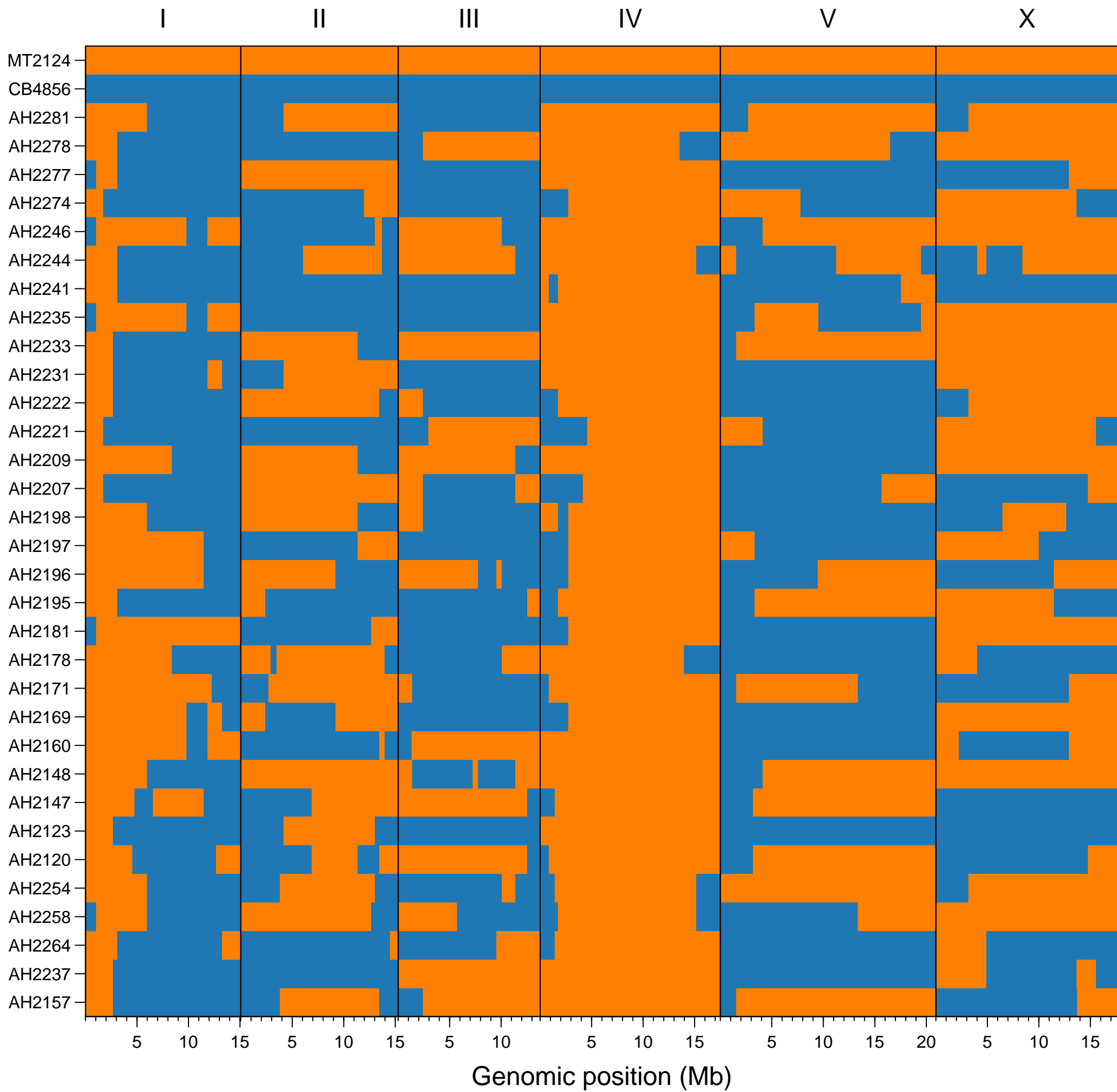

Supplement: Supplementary file 3 [file 3185FigureS2.pdf]

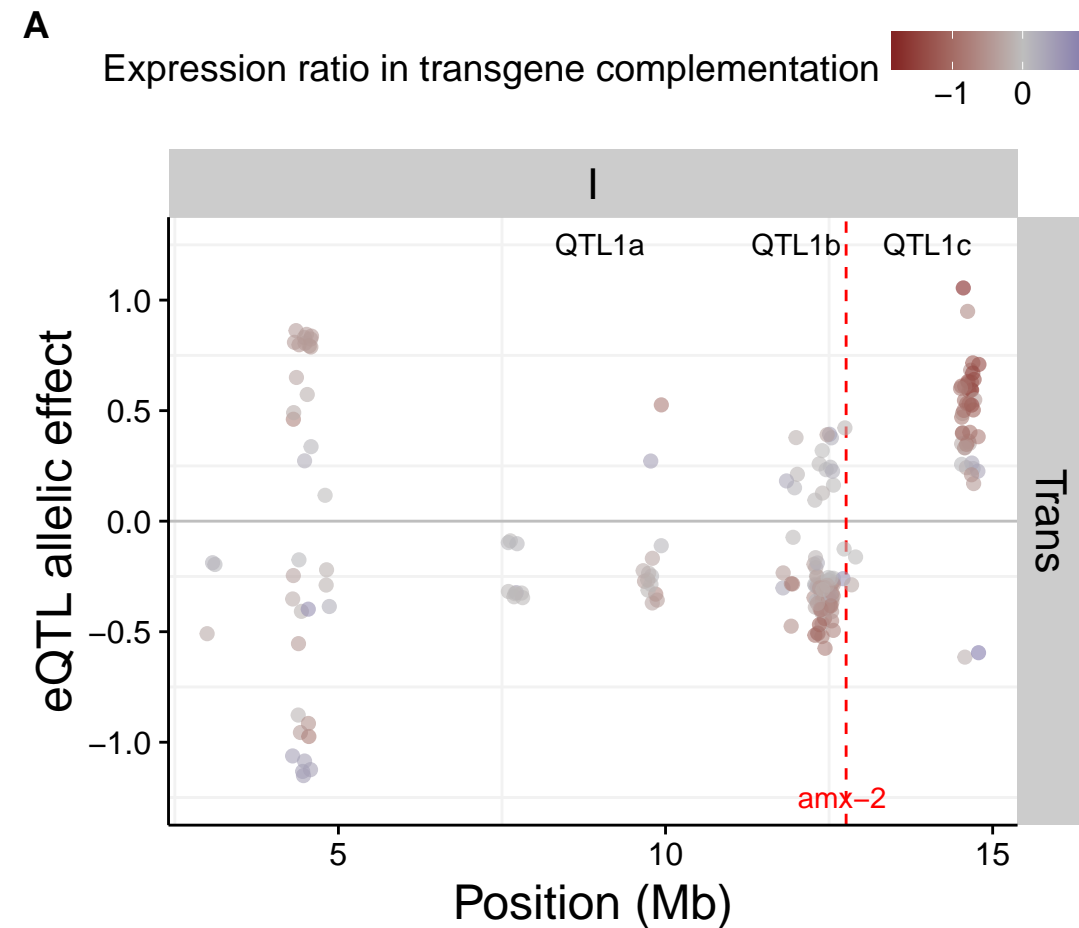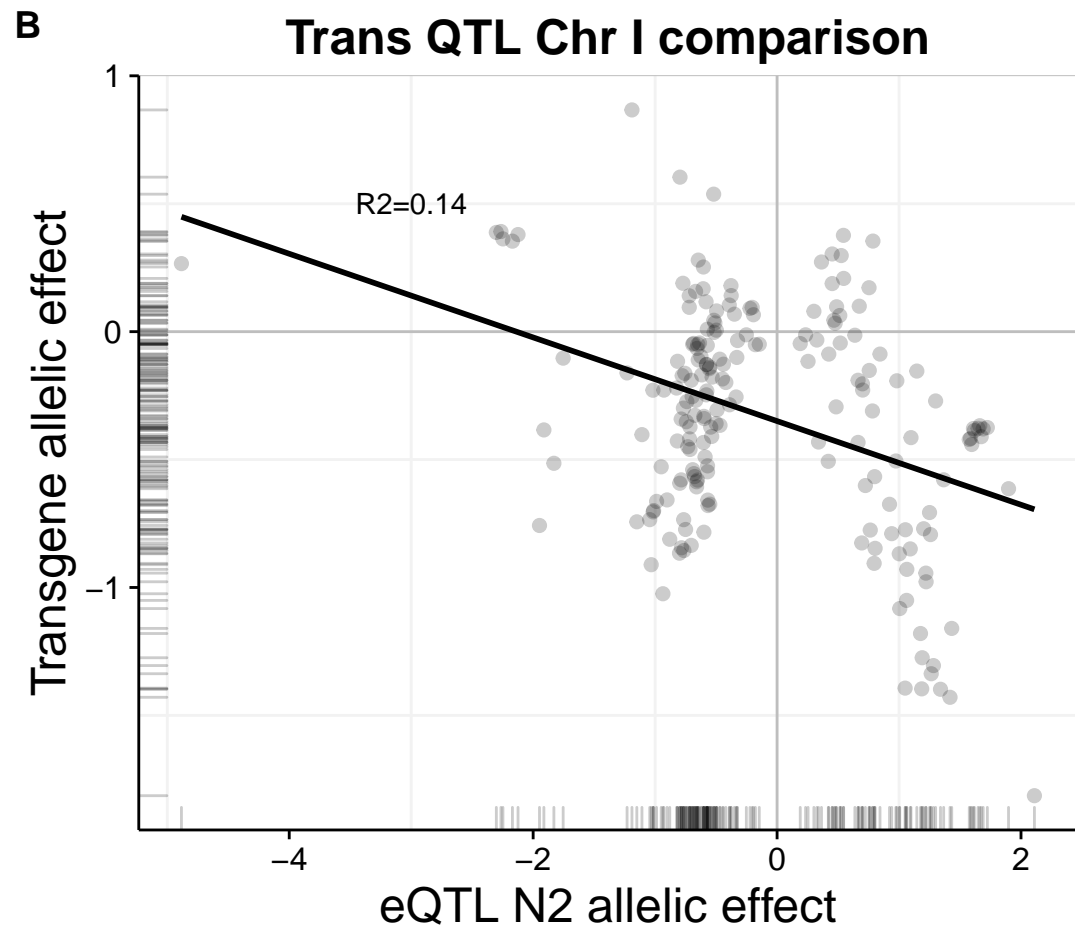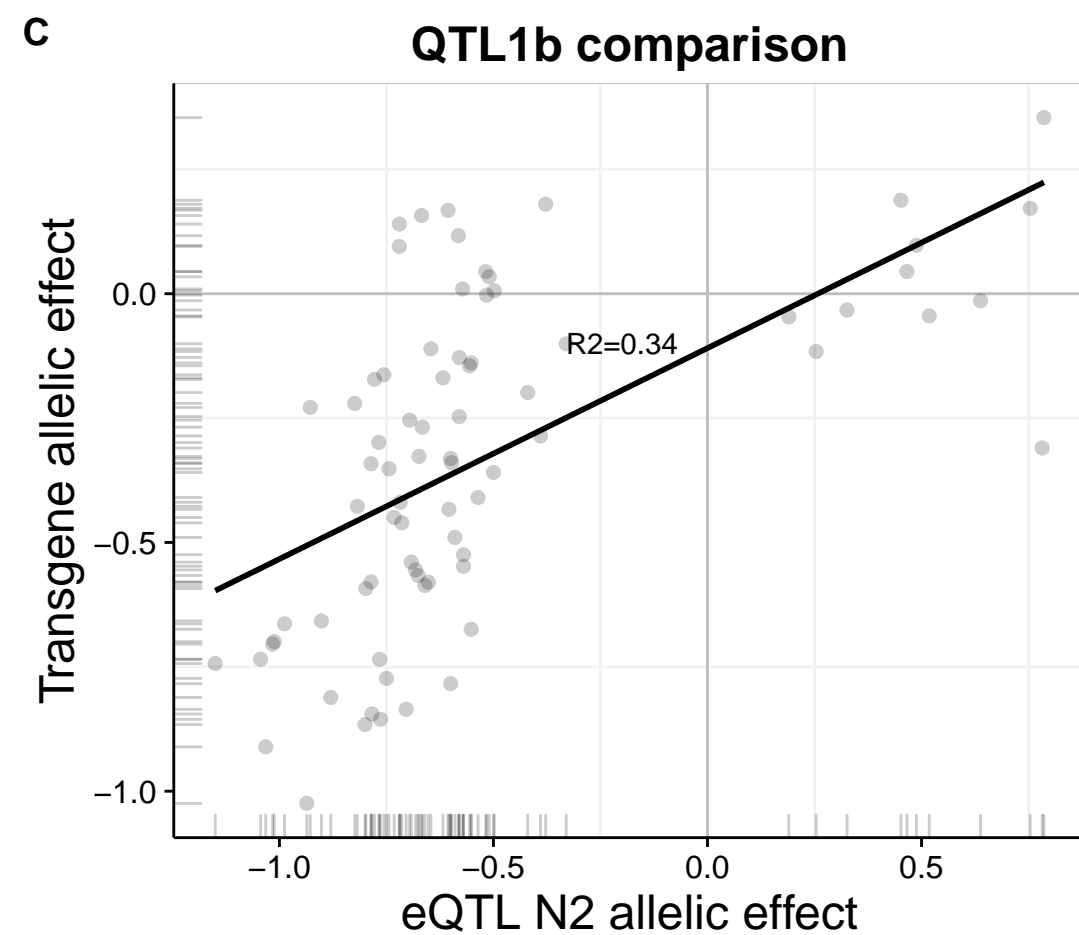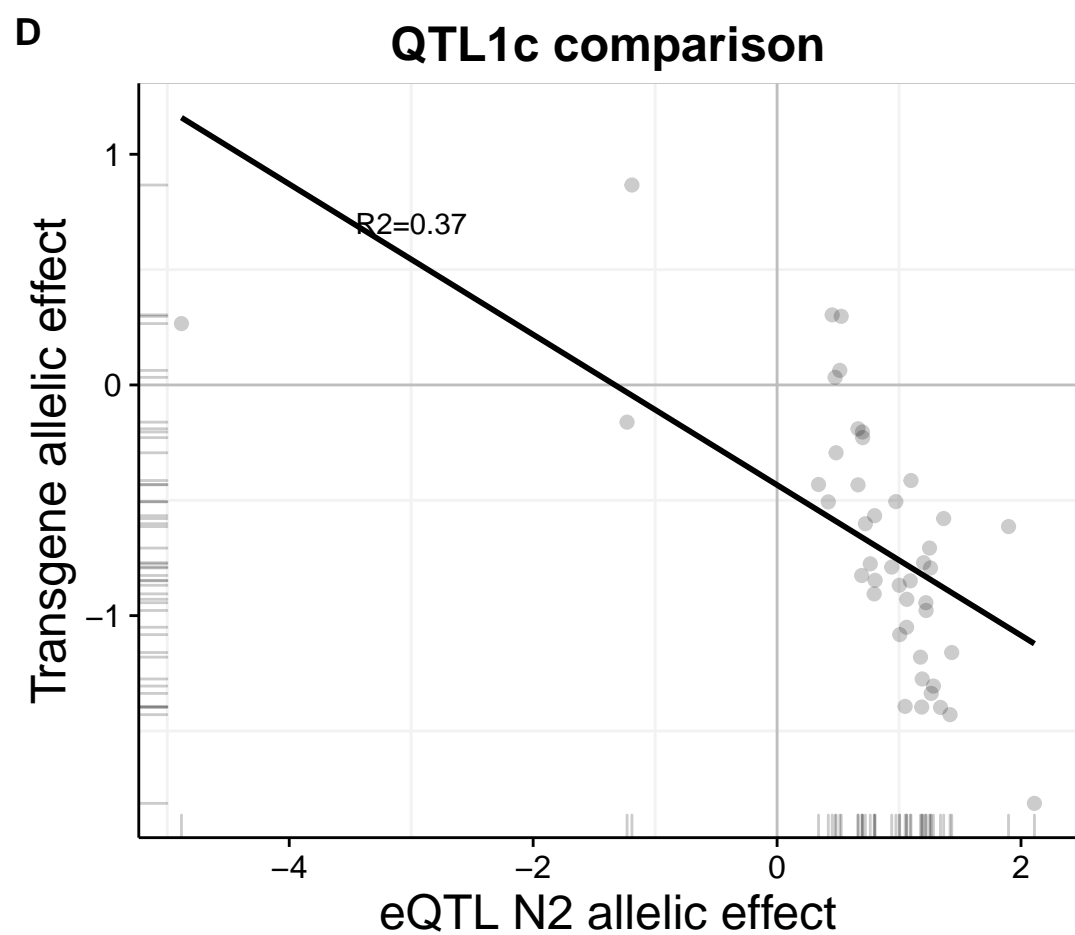

Supplement: Supplementary file 5 [file 3185FigureS4.pdf]
