# Supplementary material for: Ras/MAPK Modifier Loci Revealed by eQTL in Caenorhabditis elegans
Source: G3 (Bethesda). 2017 Jul 27;7(9):3185–93. doi: 10.1534/g3.117.1120 (PMC5592943; doi:10.1534/g3.117.1120)
Supplement: Supplementary file 7 [file 3185TableS2.docx]

**Supplemental table S2.**

The 73 fragment length polymorphisms (FLP) markers used for mapping.

*ZH1-17, ZH1-10a, ZH1-07, ZH1-18a, ZH1-03, ZH1-27, ZH1-34, ZH1-01, ZH1-23, ZH1-15, ZH1-08, ZH1-06, ZH2-04a, ZH2-16, ZH2-07, ZH2-13, ZH2-19, ZH2-02, ZH2-20, ZH2-25, ZH2-27, ZH2-09, ZH2-10, ZH2-12, ZH3-17a, ZH3-07, ZH3-06, ZH3-08, ZH3-28, ZH3-15, ZH3-04, ZH3-02, ZH3-05a, ZH3-35, ZH3-10a, ZH3-11, ZH3-13, ZH4-04a, ZH4-5, ZH4-06, ZH4-16, ZH4-08, ZH4-17, ZH4-18, ZH4-19, ZH4-20, ZH4-21, ZH4-12, ZH5-13, ZH5-03a, ZH5-14, ZH5-05, ZH5-16, ZH5-17, ZH5-18, ZH5-11, ZH5-12, ZH5-08, ZH5-21/22, ZH5-09, ZHX-17, ZHX-08, ZHX-13, ZHX-15, ZHX-10, ZHX-24, ZHX-07, ZHX-12, ZHX-11, ZHX-21a, ZHX-06, ZHX-22,* and *ZHX-23*.
